# A phase 2, multicenter, open-label study of sepantronium bromide (YM155) plus docetaxel in patients with stage III (unresectable) or stage IV melanoma

**DOI:** 10.1002/cam4.363

**Published:** 2014-12-23

**Authors:** Ragini Kudchadkar, Scott Ernst, Bartosz Chmielowski, Bruce G Redman, Joyce Steinberg, Anne Keating, Fei Jie, Caroline Chen, Rene Gonzalez, Jeffrey Weber

**Affiliations:** 1 Winship Cancer Institute, Emory UniversityAtlanta, Georgia; 2 London Regional Cancer CentreLondon, Ontario, Canada; 3 University of California-Los Angeles, Ronald Reagan UCLA Medical CenterLos Angeles, California; 4 University of Michigan Comprehensive Cancer CenterAnn Arbor, Michigan; 5 Astellas Pharmaceuticals Global DevelopmentNorthbrook, Illinois; 6 University of Colorado Denver, Comprehensive Cancer CenterDenver, Colorado; 7 H. Lee Moffitt Cancer CenterTampa, Florida

**Keywords:** Docetaxel, melanoma, survivin protein, YM155

## Abstract

Survivin is a microtubule-associated protein believed to be involved in preserving cell viability and regulating tumor cell mitosis, and it is overexpressed in many primary tumor types, including melanoma. YM155 is a first-in-class survivin suppressant. The purpose of this Phase 2 study was to evaluate the 6-month progression-free survival (PFS) rate in patients with unresectable Stage III or IV melanoma receiving a combination of YM155 plus docetaxel. The study had two parts: Part 1 established the dose of docetaxel that was tolerable in combination with YM155, and Part 2 evaluated the tolerable docetaxel dose (75 mg/m^2^) in combination with YM155 (5 mg/m^2^ per day continuous infusion over 168 h every 3 weeks). The primary endpoint was 6-month PFS rate. Secondary endpoints were objective response rate (ORR), 1-year overall survival (OS) rate, time from first response to progression, clinical benefit rate (CBR), and safety. Sixty-four patients with metastatic melanoma were treated with docetaxel and YM155. Eight patients received an initial docetaxel dose of 100 mg/m^2^ and 56 patients received 75 mg/m^2^ of docetaxel. Six-month PFS rate per Independent Review Committee (IRC) was 34.8% (*n* = 64; 95% CI, 21.3–48.6%), and per Investigator was 31.3% (*n* = 64; 95% CI, 19.5–43.9%). The best ORR (complete response [CR] + partial response [PR]) per IRC was 12.5% (8/64). The stable disease (SD) rate was 51.6% (33/64), leading to a CBR (CR + PR + SD) of 64.1% (41/64). Estimated probability of 1-year survival was 56.3%. YM155 is a novel agent showing modest activity when combined with docetaxel for treating patients with melanoma. YM155 was generally well tolerated, but the predetermined primary efficacy endpoint (i.e., 6-month PFS rate ≥20%) was not achieved.

## Introduction

Melanoma is a major health problem, with a rapidly rising incidence and mortality. Until 2011, dacarbazine and high-dose interleukin-2 were the only drugs approved for this disease; however, neither of these agents has been shown to improve median overall survival (OS) 1,2. The therapeutic landscape for this disease has changed in recent years, with the introduction of targeted and immune therapies, such as ipilimumab, pembrolizumab, vemurafenib, dabrafenib, and trametinib. These therapies recently received US Food and Drug Administration approval based on OS benefit (of note, these approvals came after the completion of the current study).

Other targeted agents are being investigated because they interfere with cell growth control and promote tumor cell death. For instance, drug candidates which are survivin suppressants are being evaluated for potential antitumor activity, such as the first-in-class small molecule YM155 3. Survivin is a member of the inhibitor of apoptosis protein family, and has been implicated in both cell survival and regulation of mitosis in cancer 4. Overexpression of survivin has been observed in many primary tumor types, including melanoma, and its expression in sentinel lymph nodes has been associated with patient outcome 5. Survivin suppression by small interfering RNA-induced spontaneous apoptosis in melanoma cells 3. These facts suggest that survivin may be a target for the treatment of melanoma (i.e., by using survivin suppressant YM155). Preclinical studies showed that YM155 suppressed both survivin protein and mRNA expression 6. Furthermore, in human studies, monotherapy with YM155 has shown modest clinical activity with a tolerable safety profile in phase 1 and 2 trials in multiple cancer types 7–10.

Survivin has been implicated in the regulation of spontaneous apoptosis rates in melanoma cells 3, and survivin suppression increases sensitivity to existing chemotherapeutic drugs and apoptotic stimuli 4,11,12. Thus, combining YM155 with a known chemotherapeutic agent may have a synergistic effect. Docetaxel is a chemotherapeutic agent that prevents mitotic spindle breakdown by stabilizing microtubule bundles. Docetaxel monotherapy has shown a low response rate (6%–17%) in patients with melanoma 13–15, and long-term treatment with docetaxel is limited because of drug resistance and side effects. In a human melanoma model, YM155 enhanced docetaxel's antitumor activity, without increasing body weight loss, suggesting that the combination of YM155 with docetaxel may be effective for the treatment of melanoma 3.

The purpose of this Phase 2 study was to evaluate the 6-month progression-free survival (PFS) rate among patients with unresectable Stage III or IV melanoma who received YM155 plus docetaxel based upon historical controls 16. This combination of treatments for melanoma was developed at a time when other effective therapies were not available; thus, at the time of its development, YM155 was a first-in-classtargeted therapy.

## Materials and Methods

### Study design

This study was an open-label, nonrandomized multicenter study (NCT01009775) supported by Astellas Pharma Global Development. The study consisted of two parts. Part 1 established the dose of docetaxel that was tolerable in combination with YM155 at 5 mg/m^2^ per day continuous infusion over 168 h every 3 weeks. Part 2 used the established docetaxel dose (75 mg/m^2^) from Part 1 to further evaluate the tolerability and activity of the combination. The primary endpoint was 6-month PFS rate. Secondary endpoints were objective response rate (ORR), 1-year OS rate, time from first response to progression, clinical benefit rate (CBR), time to response, and safety.

The protocol was approved by the appropriate institutional review boards and was conducted in accordance with the ethical principles originating from the Declaration of Helsinki and with Good Clinical Practice, as defined by the International Conference on Harmonization. All patients provided written informed consent before enrollment.

### Inclusion and exclusion criteria

Patient inclusion criteria were age ≥18 years; histologically confirmed unresectable Stage III or IV melanoma; Eastern Cooperative Oncology Group (ECOG) performance status 0 or 1; no prior systemic treatment for advanced melanoma except for adjuvant treatment; measureable disease per Response Evaluation Criteria in Solid Tumors version 1.1 (RECIST v1.1); and life-expectancy >12 weeks. Exclusion criteria included: presence or history of brain metastases, primary ocular melanoma, or mucosal melanoma; baseline neuropathy >Grade 2; and serum creatinine >1.5 times the upper limit of normal (ULN). Adequate liver and bone marrow function were also required prior to enrollment. Patients with a positive test for Hepatitis B surface antigen or Hepatitis C antibody, or with a history of a positive test for human immunodeficiency virus were excluded.

### Drug administration

**Figure 1:**
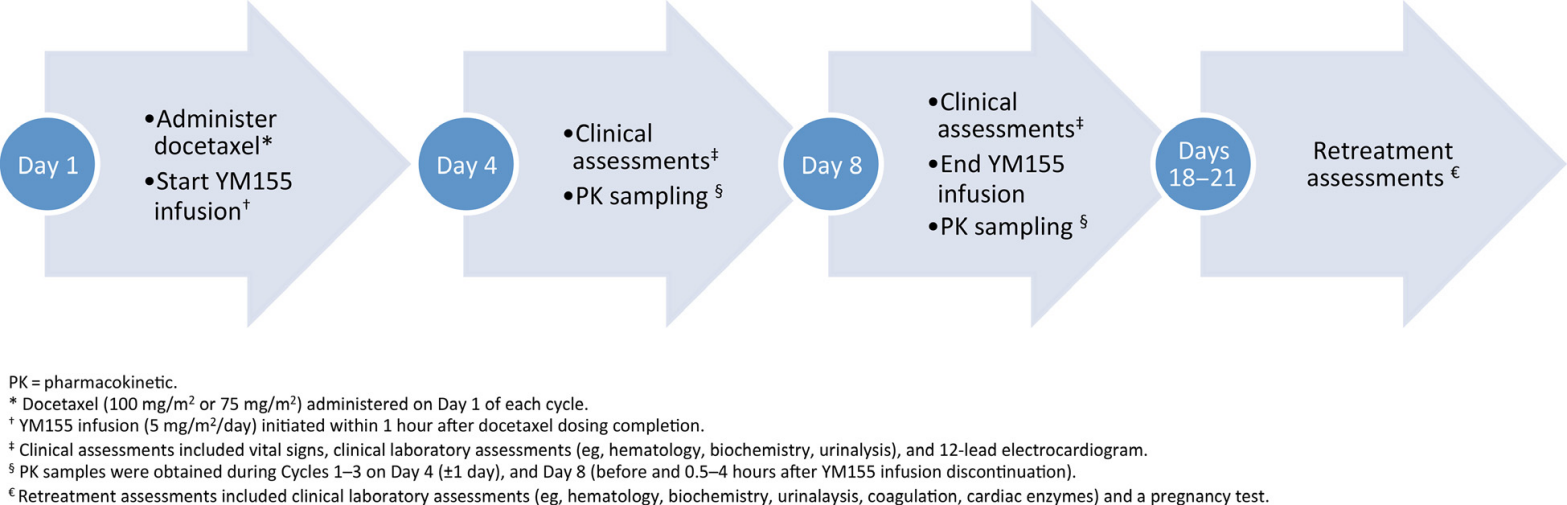
Study flow diagram.

One treatment cycle was defined as 21 days, divided into a 7-day treatment period followed by a 14-day observation period (Figure1). During Part 1, the starting dose of docetaxel was 100 mg/m^2^, administered intravenously over 1 h, but could be reduced to 75 mg/m^2^ for anticipated toxicity such as neuropathy or leukopenia; this dosing regimen was established in a previous Phase 2 trial 15. Docetaxel was administered on Day 1 of every 21-day cycle, using standard of care procedures including premedication with corticosteroids. Part 2 used the established docetaxel dose (75 mg/m^2^) from Part 1 (based on dose-limiting toxicities [DLTs]), to further evaluate the tolerability and activity of combination therapy with YM155 and docetaxel. YM155 was provided in vials, as an aqueous solution that was further diluted for administration with an appropriate volume of concentrated stock solution in 5% dextrose in water or 0.9% normal saline in a light- and temperature-controlled environment. The drug was administered at a dose of 5 mg/m^2^ per day by continuous intravenous infusion over 168 h via a portable electronic infusion pump through a central line, port, or peripherally inserted central catheter line. YM155 was administered within 1 h of completing docetaxel infusion on Day 1 of each cycle. Patients could receive the study regimen until experiencing unacceptable toxicity or disease progression, or until withdrawal of consent. Patients experiencing intolerable docetaxel toxicity were allowed to continue treatment with YM155 alone.

### Tolerability and safety evaluations

The following safety assessments were collected for each patient: subjective and objective symptoms, vital signs, laboratory tests, and 12-lead electrocardiogram. Adverse events (AEs) were graded according to the National Cancer Institute (NCI) Common Terminology Criteria for Adverse Events (CTCAE), version 4.02. DLT was assessed for patients in Part 1 of the study. A DLT was defined as any study drug-related NCI CTCAE Grade 4 hematological or Grade 2 nonhematological (excluding temporary hyperglycemia, alopecia, fatigue, and anorexia) toxicity. Specific modifications to the NCI CTCAE toxicities that are considered DLTs included the following: ANC <500/mm^3^ for >5 days; platelet count <25,000/mm^3^; serum creatinine >three times the ULN; ≥Grade 2 nausea, vomiting, or diarrhea in the presence of maximal prophylaxis; any Grade 4 liver toxicity or upward change in liver function studies of two grades for patients with preexisting liver function elevation; and/or treatment delay of >14 days.

### Pharmacokinetics and exploratory assessments

Blood samples were collected on Day 4 (±1 day), Day 8 before discontinuation of YM155, and Day 8 within 30 min to 4 h after discontinuation of YM155 during cycles 1–3 to measure plasma concentrations of YM155 and its metabolites. The samples were collected in vacuum tubes containing sodium heparin, kept chilled under ice with light protection, and centrifuged within 30 min of collection. Plasma YM155 and metabolite concentrations were measured at a bioanalytical laboratory (Pharmaceutical Product Development [PPD], Richmond, VA) using validated liquid chromatography–mass spectrometry methods. The analytes were isolated from a 0.1 mL plasma volume using solid phase extraction and quantified over an assay range of 0.05–25 ng/mL.

Exploratory endpoints included pharmacodynamic evaluation of potential biomarkers. Biomarker assessments were performed on unstained tumor tissue slides from either a historical biopsy or new biopsy for immunohistochemical (IHC) staining. IHC staining was performed, if sufficient tumor was available, to obtain information on the correlation between clinical outcome and the prevalence of resistance markers (e.g., p–glycoprotein-1, OCT–1, OCT–2) and mechanistic markers (e.g., survivin, PARP cleavage product p85, phosphorylated Erk1/2). If the amount of sample was limited, analyses were prioritized based upon significance in nonclinical studies.

Blood samples were collected before and after both docetaxel and YM155 infusions to evaluate for tumor apoptosis by M30 Apoptosense enzyme-linked immunosorbent assay (ELISA), a plasma-based test measuring a soluble cleavage product of tumor apoptosis, by means of creating a neoepitope of cytokeratin 18 (tumor-specific), during apoptotic cleavage. A Student's *t*-test analysis was performed on longitudinal serum samples to correlate M30 change from baseline with clinical response.

### Tumor assessments

Efficacy assessments were determined through results from radiological imaging and objective tumor assessments according to the requirements of RECIST v1.1. Imaging studies (i.e., diagnostic CT or MRI scans) were performed at baseline and every 6 weeks throughout the study.

### Statistical analysis

The 6-month PFS rate was defined as the probability that a patient survives without objective tumor progression at 6 months (24 weeks) after the first dose of the study regimen. Tumor progression was assessed by independent radiological review. A predetermined primary endpoint benchmark (i.e., 6-month PFS rate ≥20%) for efficacy was established, below which the combination treatment was deemed not worthy of further investigation 16. The following patients were censored at the date of the last adequate radiological assessment: patients who initiated another systemic treatment for melanoma, patients who progressed or died after missing ≥2 consecutive radiological assessments, and patients who died >89 days after last tumor scan. The full analysis set (FAS; defined as all patients who initiated ≥1 dose of YM155) was used for primary efficacy analyses. A sample size of 60 patients for Part 2 of the study was required to have 94% power to test the null hypothesis of 6-month PFS rate of 20%, using a one-sided alpha of 0.025. The 6-month PFS rate with the corresponding two-sided 95% confidence intervals (CIs) for the FAS were calculated using Kaplan–Meier estimates. The study was considered successful only if the lower boundary of the 95% CI for 6-month PFS was >20%.

Other endpoints included ORR, PFS, OS, 1-year survival rate, duration of response, CBR, and safety.

## Results

### Patients

Sixty-four patients were enrolled from December 2009 to September 2010 (Part 1, *n* = 3; Part 2, *n* = 61). During cycle 1 of Part 1, three patients received 100 mg/m^2^ of docetaxel, and experienced DLTs (e.g., reduced neutrophil count, neutropenia, febrile neutropenia) indicating the dose was not well tolerated. Per protocol instructions, a reduced dose of 75 mg/m^2^ was to be initiated for the 61 patients in Part 2, although five patients in Part 2 actually received starting doses of 100 mg/m^2^, constituting protocol violations. All protocol violations were reported to the IRB per institutional guidelines. Of the 64 patients in both parts of the study, 25 (39.1%) had their docetaxel dose reduced; all eight patients initially receiving 100 mg/m^2^ were reduced to 75 mg/m^2^, 16 patients were reduced from 75 mg/m^2^ to 55 mg/m^2^, and one patient was reduced from 75 mg/m^2^ to 60 mg/m^2^. All patients initiated YM155 at 5 mg/m^2^ per day continuous infusion over 168 h every 3 weeks along with docetaxel; seven patients received YM155 dose reductions to 3.6 mg/m^2^ per day. All enrolled patients (*n* = 64) were included in the FAS, per-protocol set, pharmacokinetics set, and safety analysis set; thus, all analyses include all enrolled patients, unless otherwise specified.

The demographic and baseline patient characteristics are listed in Table1. The population consisted of a poor prognostic group with 73.4% of the patients with M1c disease. The median (range) age was 59 (26–79) years.

**Table 1:** Baseline patient characteristics

Characteristic	Patients (*n* = 64)
Sex, *n* (%)	
Male	44 (68.8)
Female	20 (31.3)
Ethnicity, *n* (%)	
Not Hispanic or Latino	63 (98.4)
Hispanic or Latino	1 (1.6)
Age	
Median (range), years	59 (26–79)
<65 years, *n* (%)	37 (57.8)
≥65 years, *n* (%)	27 (42.2)
ECOG performance status, *n* (%)	
Grade 0	41 (64.1)
Grade 1	23 (35.9)
Metastatic (M) classification at diagnosis, *n* (%)	
M1a and M1b	17 (26.6)
M1c	47 (73.4)
Serum LDH, *n* (%)	
No data	1 (1.6)
Normal	6 (9.4)
Elevated	57 (89.1)

ECOG, Eastern Cooperative Oncology Group; LDH, lactate dehydrogenase.

### Safety

A list of all common (i.e., occurring in ≥10% of patients) AEs appears in Table2. The majority of AEs were judged by treating investigators to be Grade 1 or 2 severity, with the most common low-grade AE being fatigue (78.1%). The most clinically significant Grade 3 or 4 toxicities occurring in >5% of patients treated included neutropenia (59.4%), leukopenia (28.1%), decreased neutrophil count (25.0%), febrile neutropenia (17.2%), white blood cell count decrease (15.6%), mucositis (9.4%), fatigue (7.8%), diarrhea (6.3%), and dehydration (6.3%). Most patients (90.6%) experienced Grade 3 or 4 toxicities that were attributed to either YM155 or docetaxel. Three deaths occurred during the study; all were attributable to disease progression by the investigator.

**Table 2:** Adverse events occurring in ≥10% of patients

Adverse event, *n* (%)	Grade 1/2	Grade 3/4	All grades
Fatigue	50 (78.1)	5 (7.8)	55 (85.9)
Nausea	40 (62.5)	1 (1.6)	41 (64.1)
Decreased appetite	39 (60.9)	1 (1.6)	40 (62.5)
Neutropenia	2 (3.1)	38 (59.4)	40 (62.5)
Alopecia	37 (57.8)	0	37 (57.8)
Diarrhea	30 (46.9)	4 (6.3)	34 (53.1)
Mucositis	18 (28.1)	6 (9.4)	24 (37.5)
Pyrexia	20 (31.3)	2 (3.1)	22 (34.4)
Rash	19 (29.7)	1 (1.6)	20 (31.3)
Dysgeusia	20 (31.3)	0	20 (31.3)
Constipation	19 (29.7)	0	19 (29.7)
Leukopenia	1 (1.6)	18 (28.1)	19 (29.7)
Decreased neutrophil count	3 (4.7)	16 (25.0)	19 (29.7)
Dyspnea	17 (26.6)	1 (1.6)	18 (28.1)
Vomiting	17 (26.6)	1 (1.6)	18 (28.1)
Anemia	11 (17.2)	6 (9.4)	17 (26.6)
Headache	15 (23.4)	0	15 (23.4)
Peripheral edema	15 (23.4)	0	15 (23.4)
Arthralgia	15 (23.4)	0	15 (23.4)
Hypokalemia	11 (17.2)	3 (4.7)	14 (21.9)
Peripheral neuropathy	14 (21.9)	0	14 (21.9)
Back pain	12 (18.8)	2 (3.1)	14 (21.9)
Pain	12 (18.8)	1 (1.6)	13 (20.3)
Insomnia	12 (18.8)	0	12 (18.8)
Decreased white blood cell count	2 (3.1)	10 (15.6)	12 (18.8)
Febrile neutropenia	1 (1.6)	11 (17.2)	12 (18.8)
Hyponatremia	3 (4.7)	8 (12.5)	11 (17.2)
Stomatitis	10 (15.6)	1 (1.6)	11 (17.2)
Dehydration	7 (10.9)	4 (6.3)	11 (17.2)
Myalgia	10 (15.6)	0	10 (15.6)
Dizziness	10 (15.6)	0	10 (15.6)
Flushing	10 (15.6)	0	10 (15.6)
Hypomagnesemia	10 (15.6)	0	10 (15.6)
Cough	10 (15.6)	0	10 (15.6)
Hyperglycemia	7 (10.9)	2 (3.1)	9 (14.1)
Muscular weakness	9 (14.1)	0	9 (14.1)
Pain in extremity	9 (14.1)	0	9 (14.1)
Abdominal pain	7 (10.9)	1 (1.6)	8 (12.5)
Asthenia	7 (10.9)	1 (1.6)	8 (12.5)
Dry skin	8 (12.5)	0	8 (12.5)
Hypophosphatemia	3 (4.7)	5 (7.8)	8 (12.5)
Edema	8 (12.5)	0	8 (12.5)
Pruritus	8 (12.5)	0	8 (12.5)
Weight decreased	7 (10.9)	1 (1.6)	8 (12.5)
Anxiety	7 (10.9)	0	7 (10.9)

### Pharmacokinetic analysis

The median (range) plasma concentrations of YM155 on Day 4 during the first, second, and third infusions of YM155 were 6.510 (3.01–209.93) ng/mL, 6.070 (2.01–14.99) ng/mL, and 6.390 (3.13–489.06) ng/mL, respectively. Median YM155 concentrations in samples collected before the end of YM155 infusion on Day 8 of cycles 1, 2, and 3 were similar to the concentrations on Day 4, ranging between 6.740 ng/mL and 6.890 ng/mL; median YM155 concentrations in samples collected on Day 8 after the end of YM155 infusion were lower, ranging between 3.895 ng/mL and 4.535 ng/mL. The median concentration of two measured metabolites was below the detection limit at all time points.

### Biomarker analysis

#### Markers of apoptosis

Apoptosis was measured for 57 patients (89.1%; 9 time points per patient) by detection of a tumor-specific neoepitope of cytokeratin 18 (M30) created during apoptotic cleavage. A statistically significant increase in apoptosis from baseline was observed on Day 4 of cycles 1–3 (*P *=* *0.003, 0.008, and 0.039, respectively). There was no significant change from baseline to Day 8 of cycles 1, 2, or 3; the Day 8 values also appeared to normalize close to baseline. Best overall clinical response did not have any statistical correlation with increase in M30.

#### Markers of resistance

Fifty patients (78.1%) provided archival tumor specimens for marker analysis. Of the eight patients with partial response (PR), only six patients provided tumor specimens for evaluation; an ad-hoc logistic regression analysis found none of the biomarkers was correlated with response. Forty-five patients (70.3%) who provided tumor samples were tested for survivin staining; however, only a small number (*n* = 4) had high survivin expression levels, preventing meaningful interpretation of the association between survivin levels and disease progression.

### Efficacy

**Figure 2:**
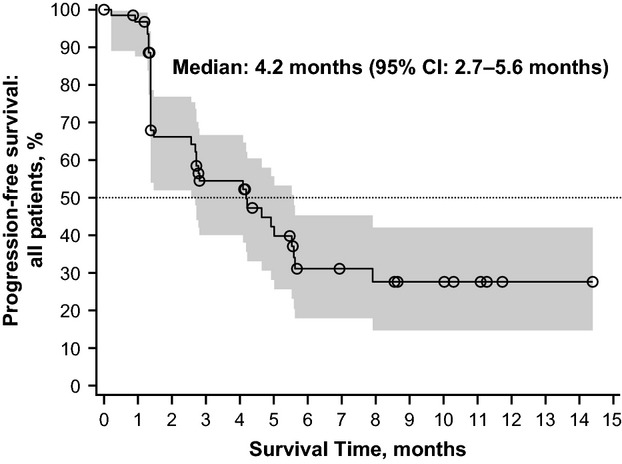
Kaplan–Meier plot with two-sided 95% confidence intervals for progression-free survival per IRC, full analysis set.

**Figure 3:**
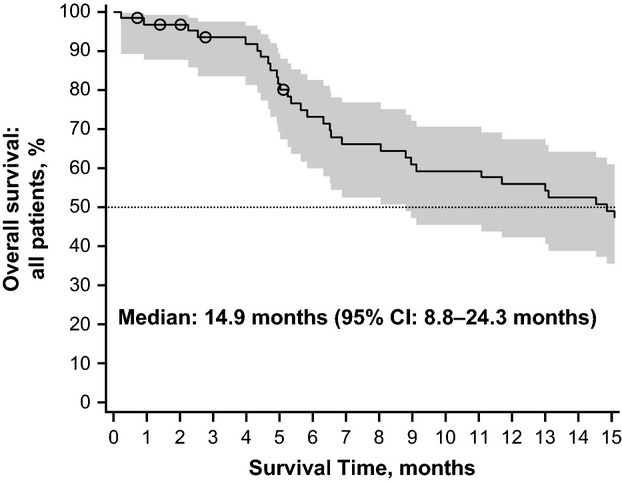
Kaplan–Meier plot with two-sided 95% confidence intervals for overall survival, full analysis set.

All efficacy analyses include all 64 enrolled patients. Data presented are from Independent Review Committee (IRC) assessment, unless otherwise specified. The median PFS was 4.2 (95% CI, 2.7–5.6) months (Figure2). Six-month PFS rate per independent assessment was 34.8% (95% CI, 21.3–48.6%); per Investigator's assessment, 6-month PFS was 31.3% (95% CI, 19.5–43.9%). The median OS was 14.9 (95% CI, 8.8–24.3) months, and the estimated probability of 1-year survival was 56.3% (Figure3).

The ORR was 12.5% (*n* = 8), with no complete responses (CRs) and eight patients (12.5%) with PR. The stable disease (SD) rate at first follow-up scan (i.e., 37 days) was 51.6% (*n* = 33), leading to a CBR (CR + PR + SD) of 64.1% (*n* = 41).

**Figure 4:**
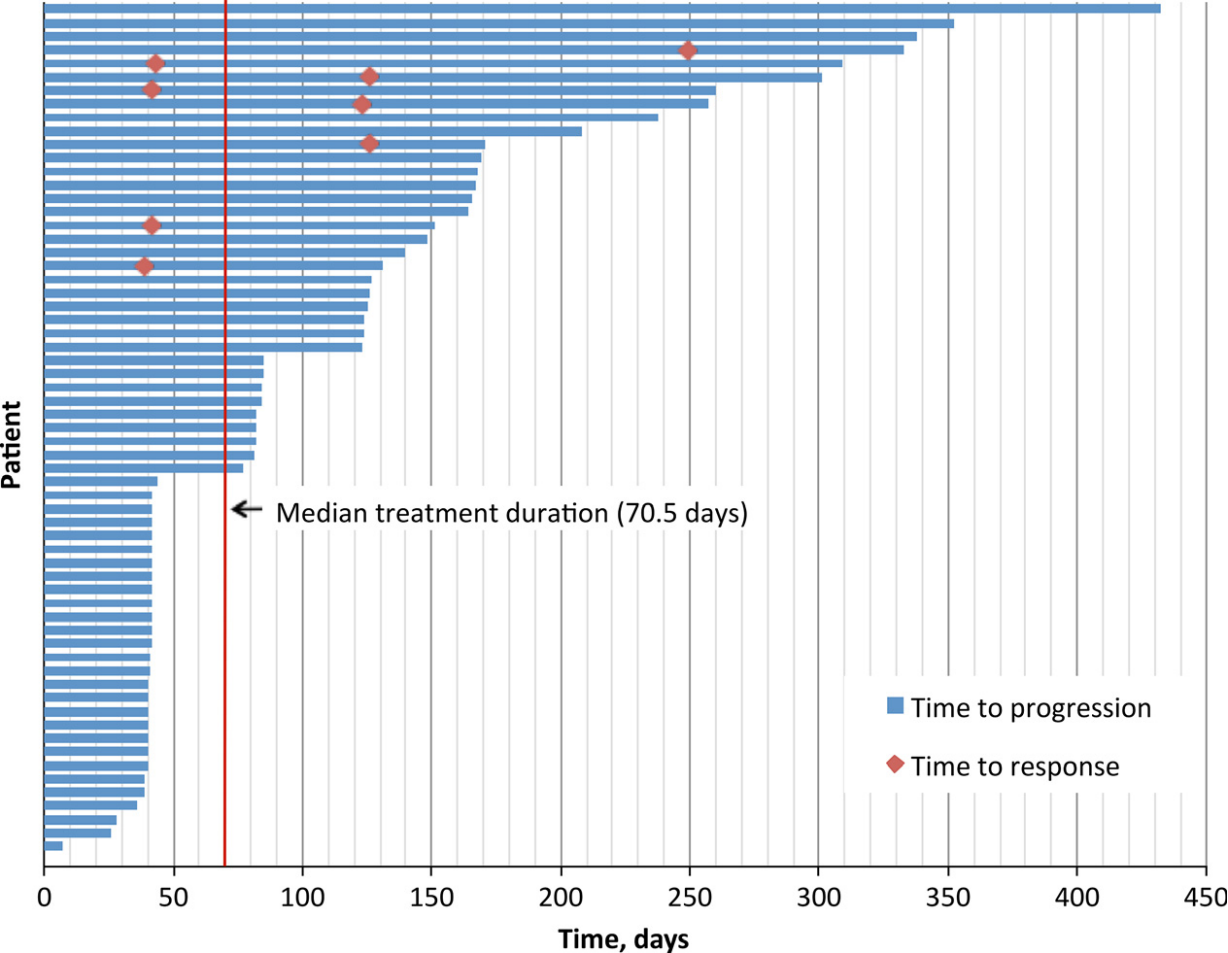
Duration of best response to YM155 by patient.

**Figure 5:**
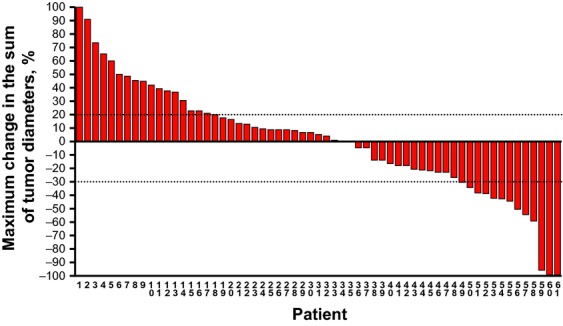
Maximum percent change in the sum of target tumor diameters by patient.

Median treatment duration was 70.5 days (Figure4). For the eight patients with PR, the median duration of response was 114 days; median duration on-study for patients having SD at first evaluation was 151 days. More patients experienced tumor diameter increase (*n* = 33; 51.6%) than decrease (*n* = 26; 40.6%), and the sum of tumor diameters decreased ≥30% in 12 patients (18.8%; Figure5).

## Discussion

YM155 is a small molecule survivin suppressant that has been evaluated for several solid tumors and lymphomas. Lewis et al. 8 first evaluated this agent in a phase 2 trial in 34 patients with metastatic melanoma, and showed that the drug was well tolerated as monotherapy. However, as a single agent, YM155 resulted in only 1 PR in the patients treated 8. Docetaxel has also demonstrated low response rates as a single agent for treating patients with melanoma 15. When considering only those patients who had maintained response or stable disease for 84 days, the CBR was 39.1% (CR, 0%; PR, 12.5% [*n* = 8]; SD, 26.6% [*n* = 17]; insufficient assessments, 23.4% [*n* = 15]).

In our study, we hypothesized that the combination of docetaxel and YM155 would enhance the efficacy of the individual agents, as was demonstrated in preclinical studies 3. This study, with an ORR of 12.5%, demonstrated higher response rates with YM155 plus docetaxel than YM155 alone 8, but not docetaxel alone 15. Although, isolated patients demonstrated significant and prolonged responses.

No increased toxicity was expected or observed due to the combination of the two drugs. The majority of patients experienced Grade 3 or 4 toxicity; however, many of the toxicities can be attributed to the anticipated effects of docetaxel (e.g., fatigue, neutropenia, febrile neutropenia, neuropathy, mucositis). Renal failure, which had been seen in previous studies of YM155 8, was not seen in this study. Most events were manageable with supportive care alone and resolved to baseline with drug interruptions or reductions.

Steady-state plasma concentrations of YM155 in this study were similar to previous monotherapy studies 17, suggesting unaltered pharmacokinetics of YM155 when administered in combination with docetaxel. The high variability observed in the plasma concentrations of YM155 was caused by outliers in the data, which could be caused by cross-contamination of blood samples with the infusion line at the time of collection.

For tumor-based IHC evaluations of resistance and mechanistic markers, no correlation was made between marker positivity and clinical outcomes for patients who had a best overall response of PR in this study. Other retrospective analyses of patients with melanoma have found that survivin upregulation was correlated with decreased survival rate, increased relapse, and chemoresistance 5,18. No correlation could be made between survivin expression levels and disease progression in this study because of the small number of samples evaluated.

YM155 with docetaxel was not associated with any unexpected tolerability concerns. The point estimate for the predetermined primary efficacy endpoint and 95% CI was achieved per IRC (i.e., 6-month PFS rate lower bound ≥20%); however, the lower bound of the 95% CI for the Investigator's assessment was below this cutoff. These data, combined with the low overall response rate, do not support further study of YM155 combined with docetaxel in patients with metastatic melanoma.

Since completion of this study, the role of targeted therapy in melanoma has rapidly expanded as many new agents have been approved. Early studies with dabrafenib and trametinib 19, pembrolizumab 20, and nivolumab and ipilimumab 21 have demonstrated notable response rates and durability of benefit. As such, the current combination of YM155 with docetaxel will not be studied further in metastatic melanoma.
